# Traumatic extremity arterial injury in children: Epidemiology, diagnostics, treatment and prognostic value of Mangled Extremity Severity Score

**DOI:** 10.1186/1749-799X-5-25

**Published:** 2010-04-15

**Authors:** Philipp Mommsen, Christian Zeckey, Frank Hildebrand, Michael Frink, Nawid Khaladj, Nadine Lange, Christian Krettek, Christian Probst

**Affiliations:** 1 Trauma Department, Hannover Medical School, 30625 Hannover, Germany; 2 Department of Cardiac, Thoracic, Transplantation and Vascular Surgery, Hannover Medical School, 30625 Hannover, Germany

## Abstract

**Background:**

Traumatic paediatric arterial injuries are a great challenge due to low incidence and specific characteristics of paediatric anatomy and physiology. The aim of the present study was to investigate their epidemiology, diagnostic and therapeutic options and complications. Furthermore, the prognostic value of the Mangled Extremity Severity Score (MESS) was evaluated.

**Methods:**

In a retrospective clinical study 44 children aged 9.0 ± 3.2 years treated for traumatic extremity arterial lesions in our Level I trauma center between 1971 and 2006 were enrolled. Exclusion criteria were age > 14, venous and iatrogenic vascular injury. Demographic data, mechanism of injury, severity of arterial lesions (by Vollmar and MESS), diagnostic and therapeutic management, complications and outcome were evaluated.

**Results:**

The most commonly injured vessel was the femoral artery (25%) followed by the brachial artery (22.7%). The mechanism of injury was penetrating (31.8%), isolated severe blunt extremity trauma (29.6%), multiple trauma (25%) and humeral supracondylar fractures (13.6%). In 63.6% no specific vascular diagnostic procedure was performed in favour of emergency surgery. Surgical reconstructive strategies were preferred (68.2%). A MESS < 7 was associated with initial (p < 0.05) and definite limb salvage (p < 0.001) of the lower extremity.

**Conclusions:**

Traumatic paediatric vascular injuries are very rare. The most common situations of vascular lesions in childhood were penetrating injuries and fractures of the extremities either as isolated injuries or in multiply injured patients. In paediatric patients, the MESS could serve as a basis for decision making for limb salvage or amputation.

## Background

Paediatric vascular injuries are rare. About 5 children with vascular lesions are treated at major U.S. trauma centers per year [1-4]. In Europe, the figures are even lower. Berqvist et al. reported 34 paediatric vascular injuries in the Swedish Vascular Registry between 1987 and 1997 [5]. Huber et al. found 26 vascular lesions in childhood over a 20-years observation period [6].

Furthermore, the study population often consists of iatrogenic and traumatic vascular injuries. Iatrogenic lesions make up one third of vascular injuries [7-9]. In addition, most authors investigate vascular injuries in children up to 18 years. In contrast, Lazarides et al. recommends focusing on children aged 13 years or younger and investigated 23 paediatric patients with arterial trauma of the extremities over a 10-years observation period [10].

Moreover, children's vascular injuries are complicated by specific characteristics of paediatric anatomy and physiology. Vascular injuries in childhood are characterized by small and thin-walled vessels with poor tissue support and the pronounced tendency to vascular spasm. Additionally, the small intravascular volume is of great importance in the treatment of paediatric vascular lesions.

In general, due to low incidence and specific anatomic and physiological characteristics, vascular injuries in children are a great challenge for the treating surgeon in terms of diagnostics, operative treatment and perioperative management.

Especially, the question of limb salvage in children with vascular injuries confronts surgeons with major problems. In 1990, Helfet et al. developed the Mangled Extremity Severity Score (MESS) for injured lower extremities in adults [11]. This scoring system provides additional prognostic information on the probability of successful permanent limb salvage with a threshold towards limb loss at a score greater than or equal to 7 [11,12]. The usefulness of the MESS in children has not been elucidated fully. Most of the available research on the MESS deals with adults, and only few data on paediatric injuries exist. Despite some reviews of paediatric open fractures indicating superior outcome in children compared to adults [13-16], there is only one retrospective study investigating the applicability of the MESS to children [17]. In the present study, besides epidemiology, diagnostics, therapeutic options and outcome of paediatric extremity arterial injuries, we investigated the prognostic value of MESS in children in order to contribute some valuable evidence to this issue.

## Methods

### Ethical approval and informed consent

The present study has been approved by the Ethical Committee of the Hannover Medical School, Germany, and has therefore been performed in accordance with the ethical standards laid down in the 1964 Declaration of Helsinki. Informed consent was obtained from all patients (or their relatives) included in the investigation.

### Inclusion and exclusion criteria

Paediatric patients with traumatic extremity arterial injuries admitted to our Level 1 trauma center between January 1971 and December 2006 were included in the present retrospective study. Further inclusion criteria were complete documentation of all required parameters for calculation of the severity scores of vascular injuries. Presence of any of the following factors led to exclusion: age > 14 years, venous and iatrogenic vascular lesions.

### Clinical Parameters

Clinical data including demographics, mechanism of injury, severity of vascular lesions (by Vollmar and MESS-Score), diagnostic and therapeutic management and complications were evaluated by a review of patient files.

Specific diagnostic procedures were defined as angiography, Doppler scan, CT angio scan. Complications were documented as being present, if there was secondary vascular occlusion, stenosis, pulse diminution or secondary haemorrhage leading to revision surgery. The development of skin dystrophies or even ulcers due to mal-perfusion was also considered to be a complication.

### Scoring systems

The assessment of severity of vascular injuries was performed according to the Score by Vollmar and the MESS.

Vollmar classified the severity of direct vascular injuries based on the lesion of the different structures of the vessel wall [18]. In blunt vascular injuries an isolated lesion of the Intima is classified as grade 1, a lesion of Intima and Media as grade 2 and a contusion of the whole vessel wall as grade 3. Penetrating vascular injuries are distributed into complete (grade 3) and partial transection (grade 2). If the vascular lumen is not opened, grade 1 is assumed.

**Figure 1:**
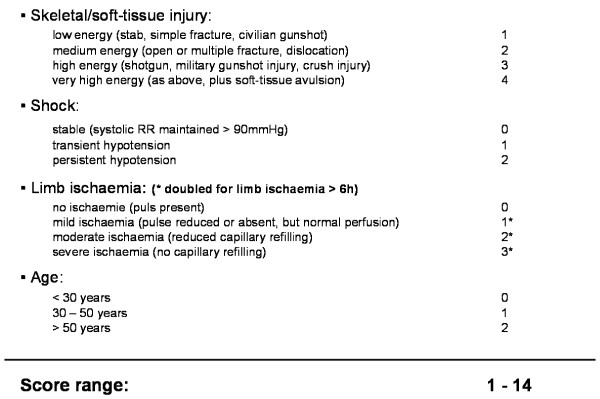
**Mangled extremity severity score (Helfet et al. 1990)**.

In 1990 Helfet et al. [11] developed the Mangled Extremity Severity Score (MESS) for injuries to the lower extremity based on 4 parameters: skeletal/soft-tissue injury, limb ischemia, shock and age (Figure 1). Shock and age are rated with a score of 0-2 each, skeletal/soft-tissue injury with a score of 1-4 and limb ischemia with a score of 0-3 (score doubled for ischemia > 6 h). Afterwards the scores of the different parameters are summed. The MESS score ranges from 1 to 14. Helfet et al. described that a MESS score greater than or equal to 7 had a 100% predictable value for amputation [11]. In a series of 164 severely injured lower limbs all cases with a score of seven ore more required amputation [12]. Therefore, the MESS seems to be accurate in discriminating between limbs that are salvageable and those that are unsalvageable and better managed by initial amputation [11,12].

### Statistical analysis

Statistical analysis was performed using SPSS statistics software program (SPSS Inc., Chicago, Illinois, USA). The level of statistical significance was set at p < 0.05. Data was subjected to the χ^2^-test or student t-test as applicable. Data are presented graphically as mean ± standard error of the mean (SEM).

## Results

### Demographics

44 children treated at our Level I trauma center between January 1971 and December 2006, aged 2-14 years (mean age 9.0 ± 3.2 years) with traumatic extremity arterial lesions were included. 35 (79.6%) patients were male and 9 female (20.4%). The average follow-up was 1.7 ± 2.5 years. Patients were initially admitted to our Level I trauma center in 52.3%, the remaining 47.7% - mostly multiply injured children or isolated severe blunt extremity injuries - were transferred.

### Mechanism and type of vascular injury

**Figure 2:**
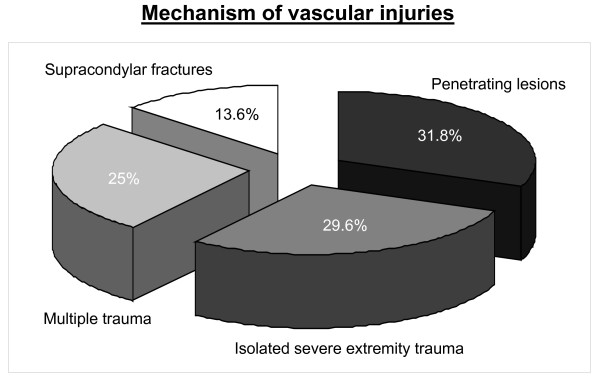
**Mechanism of vascular injuries**.

The mechanism of injury was penetrating trauma by stab or cut wounds in 31.8%, isolated severe blunt extremity trauma (29.6%), multiple trauma (25%) and supracondylar fractures of the humerus (13.6%) as demonstrated in Figure 2.

Concerning the type of injury, we found direct vascular injuries in 97.7% with penetrating lesions in 32.6% and blunt vascular trauma in 67.4%. In one case an indirect vascular injury with a rupture of the axillary artery caused by a dislocation of the shoulder joint was seen. The type and pattern of vascular injuries are presented in Table 1.

**Table 1:** Type and pattern of vascular injuries

**Indirect vascular injuries**	**1 (2.3%)**
**Direct vascular injuries**	**43 (97.7%)**
**Penetrating vascular trauma**	**14 (32.6%)**
- upper arm	3 (21.4%)
- forearm	5 (35.7%)
- thigh	2 (14.3%)
- lower leg	4 (28.6%)
**Blunt vascular trauma**	**29 (67.4%)**
open fractures	22 (75.9%)
- humerus	1 (4.5%)
- pelvis	2 (9.2%)
- femur	9 (40.9%)
- tibia	9 (40.9%)
- foot	1 (4.5%)
closed fractures	7 (24.1%)
- humerus	7 (100%) [6 × supracondylar fracture]

### Location of vascular injuries

**Figure 3:**
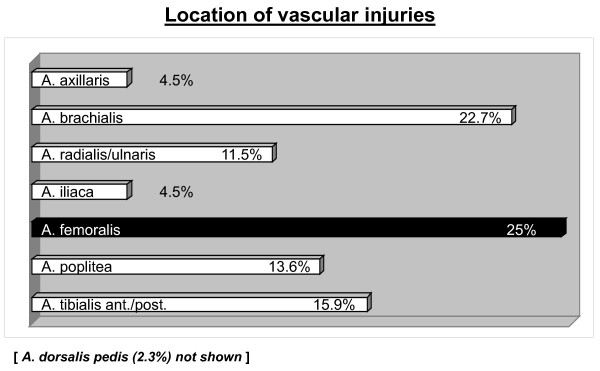
**Location of vascular injuries**.

The lower extremity was affected most frequently (61.4%) followed by the upper extremity (38.6%). The most frequently injured vessel was the femoral artery (25%) followed by the brachial artery (22.7%) as demonstrated in Figure 3.

### Diagnostics

Standard diagnostic procedures like plain x-ray of the injured extremity or whole body CT scan for polytraumatized patients by mid 1990ies were applied in all cases. In 63.6% no specific vascular diagnostic procedure was performed in favour of an emergency operation. An angiography was carried out in 20.5% and a colour Doppler scan in 11.4%. In 4.5% both diagnostics were applied. Follow up diagnostics consisted of clinical examination. In case of suspected thrombosis or stenosis Doppler scan was performed followed by CT angio scan.

### Treatment

Concerning the operative treatment of vascular injuries, surgical reconstructive procedures like direct vascular repair, interposition grafts and vascular patches were preferred (30 patients, 68.2%). Vascular ligation (3 patients, 6.8%) or bypass surgery (1 patient, 2.3%) were uncommon procedures. An initial amputation of the affected extremity was performed in 3 patients. In 15.9% an adequate vascular perfusion was achieved after fracture reduction and therefore no specific vascular surgery was required. In general, adequate vascular perfusion was assessed by pulse examination and Doppler scan. A concomitant dermato-fasciotomy was performed in 7 patients (15.9%). No patient developed a compartment syndrome after the operative treatment. Further data are shown in Table 2. Regarding postoperative anticoagulation, pharmacological treatment protocols were performed individually in every patient depending on age, severity of vascular lesions and accompanying injuries. In general, there are no guidelines for anticoagulation after paediatric vascular injuries. Basically, the same standards should be applied as in adults containing low-dose heparin and platelet aggregation inhibitor for 3 months.

**Table 2:** Diagnostics and surgical treatment of vascular injuries

				Total	Penetrating	Isolated severe	Multiple	Supracondylar
		
					injuries	extremity trauma	trauma	fractures
**Number of participants**				44	14 (31.8%)	13 (29.6%)	11 (25%)	6 (13.6%)

**Male:female**				35:9	12:2	11:2	9:2	3:3

**Age (years)**				9.0 ± 3.2	9.9 ± 2.5	7.9 ± 3.7	9.0 ± 3.2	8.0 ± 3.0

**MESS**				5.0 ± 2.5	3.5 ± 2.0	5.5 ± 2.3	7.0 ± 2.4	3.7 ± 1.6

**Diagostics:**								

Clinical examination/ emergency surgery				28 (63.6%)	11 (78.6%)	8 (61.5%)	3 (27.3%)	6 (100%)

Angiography				9 (20.5%)	0 (0%)	4 (30.8%)	5 (45.4%)	0 (0%)

Doppler Scan				5 (11.4%)	2 (14.3%)	1 (7.7%)	2 (18.2%)	0 (0%)

Both				2 (4.5%)	1 (7.1%)	0 (0%)	1 (9.1%)	0 (0%)

**Surgical Treatment:**								

Primary repair/interposition graft/patch				30 (68.2%)	12 (85.7%)	7 (53.8%)	9 (81.8%)	2 (33.3%)

Vascular Ligation				3 (6.8%)	2 (14.3%)	1 (7.7%)	0 (0%)	0 (0%)

Vascular Bypass				1 (2.3%)	0 (0%)	1 (7.7%)	0 (0%)	0 (0%)

Primary Amputation				3 (6.8%)	0 (0%)	2 (15.4%)	1 (9.1%)	0 (0%)

Fracture reduction (no specific vascular surgery)				7 (15.9%)	0 (0%)	2 (15.4%)	1 (9.1%)	4 (66.7%)

**Limb salvage (initial)**				41 (93.2%)	14 (100%)	11 (84.6%)	10 (83.3%)	6 (100%)

**Limb salvage (definite)**				36 (81.8%)	13 (92.9%)	9 (69.2%)	8 (72.2%)	6 (100%)

### Complications

In 81.8% no post operative complications were found. Vascular thrombosis or stenosis and secondary peripheral ulcers could be observed in 13.6% and 4.5% respectively. No secondary haemorrhage requiring revision surgery was noted in our patients. In the present study 3 patients (6.8%) died during their hospital stay. All non-survivors were patients with multiple trauma.

### Severity of vascular injuries, outcome and prognostic value of MESS

**Figure 4:**
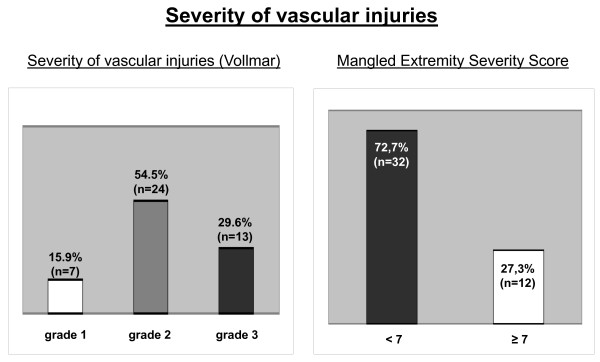
**Severity of vascular injuries**.

**Figure 5:**
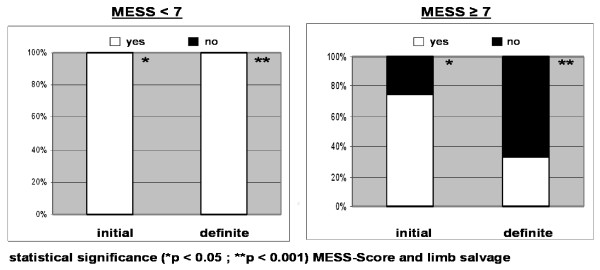
**Association of MESS and limb salvage of the lower extremity**.

The mean MESS was 5.0 with a range from 1 to 12 points. Figure 4 shows the distribution of the vascular severity grading by Vollmar and the MESS. Initial limb salvage was performed in 41 patients (93.2%) and definite limb salvage was achieved in 36 patients (81.8%). Depending on the mechanism of injury, there are significant differences between patients concerning MESS and limb salvage. According to the MESS, vascular lesions in isolated blunt extremity trauma (5.5 ± 2.3) and multiple trauma (7.0 ± 2.4) were much more severe than arterial injuries caused by penetrating lesions (3.5 ± 2.0) and supracondylar fractures (3.7 ± 1.6). Therefore, limb salvage was more often achieved in penetrating lesions and supracondylar fractures (Table 2). Furthermore, there were significant differences between upper and lower extremity injuries between patients concerning MESS and limb salvage. With an average MESS of 3.3 ± 1.4 vs. 6.1 ± 2.6 injuries of the lower extremity were much more severe than lesions of the upper extremity (p < 0.05). Accordingly, there was no MESS ≥ 7 in patients with an injury of the upper extremity. The initial and definite limb salvage of the upper extremity (n = 17) was achieved in all patients. Concerning the lower extremity (n = 27), there was a significant association of initial (p < 0.05) and definite (p < 0.001) limb salvage and MESS (Figure 5). In all patients with a MESS < 7 (n = 15), the lower extremity was salvaged. In contrast, patients with a MESS ≥ 7 of the lower extremity (n = 12) underwent initial amputation in 25% (n = 3). A definite salvage of the lower extremity was achieved in 33.3% (n = 4) when the MESS was greater than or equal to 7.

## Discussion

Due to low incidence and specific anatomic and physiological characteristics, vascular injuries in children are a great challenge in terms of diagnostics, operative treatment and perioperative management. Furthermore, the question of limb salvage in children with vascular injuries confronts surgeons with major problems. The aim of the present study was to investigate the epidemiology, diagnostic and therapeutic options and complications in traumatic extremity paediatric vascular injuries and to evaluate the prognostic value of the Mangled Extremity Severity Score (MESS). The major findings were that 1) traumatic extremity paediatric vascular injuries are very rare even at a Level I trauma center, 2) that the most common situations of traumatic vascular lesions in childhood were penetrating injuries and fractures of the extremities either as isolated injuries or in multiply injured patients, and 3) that the MESS could serve as a basis for decision making for limb salvage or amputation in paediatric patients.

We are aware that our study has some limitations. One of the most important limitations is the study design as a retrospective review of a consecutive case series over a long time period. Due to the low incidence of paediatric vascular injuries [1-6], a long observation span is always needed in order to create an adequate study population. In the present study only traumatic arterial lesions were enrolled. Therefore venous and iatrogenic vascular injuries were excluded. Because an age ≤ 14 years is more adequate for a paediatric population children older than 14 years were also excluded. Although an additional long observation period is caused, these exclusion criteria make the present study very unique. Most of the current studies investigate mixed patient populations of children and young adults aged up to 18 years with iatrogenic and traumatic injuries [8]. In contrast, Lazarides et al. observed vascular injuries of the extremities over a 10-years study period in children aged 13 years or younger [10]. But again, iatrogenic as well as traumatic arterial injuries were included in this study [10]. Bearing in mind that iatrogenic lesions make up one third of vascular injuries [7-10] our study represents a large series of 44 traumatic arterial injuries.

In accordance to the current literature [19-21], the most common situations of paediatric vascular lesions in the present study were penetrating injuries (31.8%) and either isolated fractures of the extremities or in polytraumatized patients. With 34.8% penetrating lesions Lazarides et al. reported comparable figures [10]. Due to a high rate of gun shot wounds (70.8%) penetrating vascular injuries were observed much more often (91.7%) in a study of De Virgilio et al. at a major U.S. trauma center [1]. In our study there were no gun shot injuries. The vast majority was due to stab and cut wounds. Humeral supracondylar fractures with vascular lesions were rare in our study population (13.6%). The incidence of neurovascular complications in supracondylar fractures is up to 24% [22,23]. Due to good vascular collateralisation at the upper extremity [24,25], an obstruction of the brachial artery sometimes does not become clinically apparent. In a series of 143 supracondylar fractures Shaw et al. reported an ischemia at presentation in 12% [26]. A remaining ischemia after fracture reduction was observed in three cases (2.1%). In general, persistent ischemia after reduction of supracondylar fractures is rare [27-29]. Accordingly, in the present study an adequate vascular perfusion was achieved after fracture reduction in 66.7% of supracondylar fractures. Good vascular collateralisation and commonly achieved vascular reperfusion after fracture reduction maybe explain why vascular injuries are often not registered in supracondylar fractures.

In accordance to De Virgilio et al., who reported an affection of the lower extremities in 64.6% and the upper extremity in 35.4% [1], in the present study the lower extremity was affected most frequently followed by the upper extremity. Lazarides et al. observed an almost equal distribution between upper (56.5%) and lower extremity (43.5%) [10]. In contrast to the current literature [10,30], in our study population the brachial artery (22.7%) was affected less frequently than the femoral artery (25%) caused by the lower incidence of supracondylar fractures.

In the present study, most patients (63.6%) received no specific vascular diagnostics, especially patients with supracondylar fractures, penetrating injuries and isolated blunt extremity trauma. In contrast, vascular diagnostics were performed in 72.7% of multiple trauma patients. Because of subsequent potential deterioration in multiple trauma patients, the preoperative examination of vascular lesions might be of special interest in order to avoid long surgical procedures with intraoperative evaluation of vascular injuries. In penetrating injuries and isolated blunt extremity trauma the danger of subsequent deterioration due to the second hit of the operative procedure is negligible. Furthermore, routine surgical revision of the soft tissues is required and the intraoperative examination of vascular lesions is probably easily performed. This might explain the higher rate of vascular diagnostics in multiple trauma patients. The fact that no vascular diagnostics were performed in supracondylar fractures could be explained by the frequent clinical inapparence at the time of admission as described above [24-26].

Compared to current studies, which report limb salvage rates of 87-100% in paediatric vascular injuries [1,10], a limb salvage was achieved less frequently (81.8%) in the present study. Due to the exclusion of venous and iatrogenic lesions, the severity of vascular injuries might be higher in our study population explaining the lower limb salvage rate. This might be also the explanation for the higher rate of postoperative complications with vascular occlusion (13.6%) and secondary peripheral ulcers (4.5%) compared to a study of Lazarides et al. who observed none of these complications after surgical repair or medical treatment of 23 children with arterial trauma of the extremities [10]. In a series of 550 adult patients with traumatic lower limb arterial injuries Hafez et al. reported a failure rate of 8% after surgical vascular repair indicating the more sophisticated surgical procedures in children [31].

The prognostic value of the MESS in children remains questionable as most of the available studies dealt with adults. A 100% predictable value of a MESS score greater than or equal to 7 for amputation is described in adults [11,12]. Bosse et al. reported in a series of 556 lower extremity trauma a definitive limb salvage of 34.6% in adults with MESS ≥ 7 and 82.1% in patients with MESS < 7 [32]. Few data on paediatric injuries are available. Besides some reviews of paediatric open fractures [13-16], there is one retrospective investigation focussing on the relevance of the MESS in 36 children with grade IIIB and IIIC open lower extremity fractures [17]. Fagelman et al. reported a limb salvage of 28.6% in patients with a MESS ≥ 7 and 89.7% in patients with a MESS < 7, respectively [17].

In the present study, we found comparable limb salvage rates of the lower extremity (n = 27). Definitive limb salvage was achieved in 33.3%, when the MESS was greater than or equal to 7, whereas the affected extremity could be salvaged in 100% in children with a MESS < 7. Unlike adults, in whom initial amputation rates of 43-46% are reported [12,32], in our study a primary amputation in children with a MESS ≥ 7 was performed less frequently (25%). In summary, the MESS could serve as a basis for prediction of limb salvage in children. But it has to be pointed out, that according to our results in one third of the children with a MESS ≥ 7 a limb salvage could be achieved. Therefore, the decision for limb salvage or primary amputation has to be made individually. Furthermore, the present study is limited by the number of patients and its retrospective design. Especially, the retrospective application of the MESS to the treatment of paediatric vascular injuries before the development of the score in 1990 is a weakness. Moreover, in the MESS age is not really pertinent as it remains a constant. Additionally, many of the included children were managed before modern diagnostic and therapeutic methods were developed. Advances in imaging and operative treatment of vascular and soft tissue injuries have undoubtedly influenced limb outcomes after trauma. In a prospective study with an increased MESS threshold for primary amputation (MESS ≥ 10), Lin et al. reported a successful limb salvage in 75% [33]. In general, further studies analysing a larger patient population by prospective - preferably randomized controlled - study design are required in order to validate the results of the present study.

## Conclusions

Traumatic paediatric vascular injuries are rare, even in a large Level I trauma center. The most common situations of vascular lesions in childhood were penetrating injuries and either isolated fractures of the extremities or extremity injuries in polytraumatized patients. Supracondylar fractures with vascular lesions were rare in our study population. Initial reconstructive surgery was by far the most common treatment strategy for our patients, even though sophisticated surgical technique is required. Furthermore, in our retrospective evaluation of paediatric patients, the MESS seems to be suitable to aid in decision making for limb salvage or amputation. Further studies analysing a larger patient population by prospective - preferably randomized controlled - study design are required in order to validate the results of the present study.

## Competing interests

The authors declare that they have no competing interests.

## Authors' contributions

All authors have made substantial contributions to conception and design of the study, acquisition of data, analysis and interpretation of data, drafting the article and revising of the article for important intellectual content. All authors have read and approved the final manuscript.

## References

[B1] De Virgilio C Mercado PD Arnell T Noniatrogenic pediatric vascular trauma: a ten-year experience at a Level I trauma center Ann Surg 1997 63/9 781 4 9290521

[B2] Meagher DP Defore WW Mattox KL Vascular trauma in infants and children J Trauma 1979 19 7 532 6 458896

[B3] Reed MK Lovury PA Myers SI Successful repair of pediatric popliteal artery trauma Ann J Surg 1990 160 3 287 90 10.1016/S0002-9610(06)80025-5 2393057

[B4] Reichard KW Reyes HM Vascular trauma and reconstructive approaches Semin Pediatr Surg 1990 3 2 124 32 8062056

[B5] Bergqvist D Karacagil S Westmann B Paediatric arterial trauma Eur J Surg 1998 164 10 723 31 10.1080/110241598750005345 9840300

[B6] Huber R Müller BT Ernst S Besonderheiten von Gefäßverletzungen im Kindesalter Gefässchirurgie 2004 9 117 21 10.1007/s00772-004-0336-5

[B7] Flanigan DP Keifer TJ Schuler JJ Experience with iatrogenic pediatric vascular injuries. Incidence, etiology, management and results Ann Surg 1983 198 4 430 42 10.1097/00000658-198310000-00003 6625714 PMC1353180

[B8] Gutschi S Koter H Justich E Iatrogene Gefäßverletzungen im Kindesalter Zentralbl Chir 1984 109 13 854 8 6475361

[B9] Lin PH Dodson TF Bush RL Surgical intervention for complications caused by femoral artery catherization in pediatric patients J Vasc Surg 2001 34 6 1071 8 10.1067/mva.2001.119043 11743563

[B10] Lazarides MK Georgiadis GS Papas TT Operative and nonoperative management of children aged 13 years or younger with arterial trauma of the extremities J Vasc Surg 2006 43 1 72 6 10.1016/j.jvs.2005.09.031 16414390

[B11] Helfet DL Howey T Sanders R Limb salvage versus amputation. Preliminary results of the Mangled Extremity Severity Score Clin Orthop Relat Res 1990 256 80 6 2194732

[B12] Robertson PA Prediction of amputation after severe lower limb trauma J Bone Joint Surg Br 1991 73 5 816 8 1894673 10.1302/0301-620X.73B5.1894673

[B13] Bartlett CS Weiner LS Yang E Treatment of type II and type III open tibia fractures in children J Orthop Trauma 1997 11 357 62 10.1097/00005131-199707000-00010 9294800

[B14] Blasier R Barnes C Age as a prognostic factor in open tibial fractures in children Clin Orthop 1996 331 261 4 10.1097/00003086-199610000-00037 8895648

[B15] Cullen MC Roy DR Crawford AH Open fracture of the tibia in children J Bone Joint Surg Am 1996 78 1039 47 8698721 10.2106/00004623-199607000-00008

[B16] Grimard G Naudie D Laberge LC Open fractures of the tibia in children Clin Orthop 1996 332 62 70 10.1097/00003086-199611000-00009 8913146

[B17] Fagelmann MF Epps HR Rang M Mangled Extremity Severity Score in children J Pediatr Orthop 2002 22 2 182 4 10.1097/00004694-200203000-00009 11856926

[B18] Vollmar J Rekonstruktive Chirurgie der Arterien 1996 Stuttgart, Germany: Thieme

[B19] Kioumehr F Yaghmai I Bakody P Delayed common femoral artery stenosis due to blunt trauma J Can Assoc Radiol 1989 40 324 5 2598080

[B20] Mills RP Robbs JV Paediatric arterial injury: management options at the time of injury J R Coll Surg Edinb 1991 36 13 7 2037991

[B21] O'Neill JA O'Neill JA Traumatic vascular lesions in infants and children Vascular disorders of childhood 1983 Philadelphia: Lea and Febiger 181 93

[B22] Riches KJ James RA Gilbert JD Fatal childhood vascular injuries associated with seat belt use Ann J Forensic Med Pathol 2002 23 1 45 7 10.1097/00000433-200203000-00009 11953493

[B23] Wynsma LA Negative outcomes of intravascular therapy in infants and children AACN Clin Issues 1998 9 1 49 63 9505572 10.1097/00044067-199802000-00005

[B24] Garbusz DS Leitch K Wright JG The treatment of supracondylar fractures in children with an absent radial pulse J Pediatr Orthop 1996 16 5 594 6 8865043 10.1097/00004694-199609000-00009

[B25] Kumar R Trikha V Malhotra R A study of vascular injuries in pediatric supracondylar humeral fractures J Orthop Surg (Hong Kong) 2001 9 2 37 40 12118129 10.1177/230949900100900208

[B26] Shaw BA Kasser JR Emans JB Management of vascular injuries in displaced supracondylar humerus fractures without arteriography J Orthop Trauma 1990 4 25 9 2313426 10.1097/00005131-199003000-00004

[B27] Louahem DM Nebunescu A Canavese F Neurovascular complications and severe displacement in supracondylar humerus fractures in children: defensive or offensive strategy? J Ped Ortho 2006 15 1 51 7 10.1097/01202412-200601000-00011 16280721

[B28] Rasool MN Naidoo KS Supracondylar Fractures: Posterolateral Type with Brachialis Muscle Penetration and Neurovascular Injury J Ped Ortho 1999 19 4 518 22 10.1097/00004694-199907000-00019 10413005

[B29] Sabharwal S Tredwell SJ Beauchamp RD Management of Pulseless Pink Hand in Pediatric Supracondylar Fractures of Humerus J Ped Ortho 1997 17 3 303 10 10.1097/00004694-199705000-00007 9150016

[B30] Roth S Schulte S Horsch S Arterielle und venöse Gefäßverletzungen Allgemeine und Viszeralchirurgie in up2date 2008 3 193 212 10.1055/s-2008-1038585

[B31] Hafez HM Woolgar J Robbs JV Lower extremity arterial injury: Results of 550 cases and review of risk factors associated with limb loss J Vasc Surg 2001 33 1212 9 10.1067/mva.2001.113982 11389420

[B32] Bosse MJ MacKenzie EJ Kellam JF A prospective evaluation of the clinical utility of the Lower-Extremity-Injury-Severity Scores JBJS 2001 83A 1 3 14 10.2106/00004623-200101000-00002 11205855

[B33] Lin CH Wei FC Levin LS The functional outcome of lower-extremity fractures with vascular injury J Trauma 1997 43 480 5 10.1097/00005373-199709000-00015 9314311

